# Myotonic Dystrophy: An Anaesthetic Dilemma

**Published:** 2009-12

**Authors:** N Gupta, K N Saxena, Asish Kumar Panda, Raktima Anand, Anil Mishra

**Affiliations:** 1 Assistant Prof.,Department of Anaesthesia & Intensive Care, PGIMER and associated RML hospital; 2 Professor; 3 P.G.Student; 4 Director, Professor & HOD; 5 Medical Officer, Department of anaesthesia, Intensive care & Perioperative medicine, Maulana Azad Medical College , Delhi Gate, New Delhi- 110002

**Keywords:** Anaesthesia, Myotonic dystrophy, Postoperative complication

## Abstract

**Summary:**

Myotonic dystrophy (dystrophia myotonica, DM) is a chronic, slowly progressing, highly variable inherited multisystemic disease that can manifest at any age from birth to old age. We present a 32-year-old female with adenexal mass posted for exploratory laparotomy. She was a known case of dilated cardiomyopathy (DCMP).The ECG suggested incomplete RBBB & LAHB & the ECHO revealed mild mitral regurgitation, tricuspid regurgitation, pulmonary artery hypertension with severe left ventricular dysfunction (ejection fraction of 30-35%). General anaesthesia (GA) with epidural anaesthesia was planned. The patient was haemodynamically stable through out the surgical procedure. The patient was reversed and shifted to post anaesthesia care unit. On the 2nd postoperative day patient developed respiratory distress and hypotension. ABG revealed Type 1 respiratory failure. Since the patient didn't improve with oxygen therapy and nebulisation, she was intubated and shifted to ICU. Patient was tolerating the tube without sedation and relaxants so, consultant anaesthesiologist asked for neurologist referral to rule out myotonic dystrophy. Subsequent muscle biopsy and genetic analysis was suggestive of myotonic dystrophy. Despite all possible efforts we were unable to wean her off the ventilator for 390 days. Patients with myotonic dystrophy are a challenge to the attending anaesthesiologist. These patients can be very well managed with preoperative optimized medical treatment and well-planned perioperative care.

## Introduction

Myotonic dystrophy is a multisystem disorder involving skeletal, smooth and cardiac muscle and presents with serious anaesthetic problems. The disease manifests in early childhood with myotonia, progressive weakness and wasting of muscles of face, sternocleidomastoids (SCM), distal extremities and respiration. Extra muscular features include cataracts, cardiomyopathy, conduction abnormalities, restrictive lung disease, central and obstructive sleep apnoea syndrome, dysphagia, delayed gastric emptying and endocrine abnormalities such as hypothyroidism, primary hypogonadism, infertility and diabetes mellitus^1^,^2^.

## Case report

A 32-year-old female, weight 46 kg, para 2, live children 0, with adenexal mass (suspected malignant ovarian tumor) was scheduled for exploratory laparotomy. On preanaesthetic appraisal it was found that she was a diagnosed case of dilated cardiomyopathy (DCMP). She had upper respiratory tract infection for past 3 weeks for which she was on antibiotics. She also gave history of sudden oxygen desaturation while undergoing colonoscopy for evaluating metastatic bowel involvement under 3mg iv midazolam. The routine investigations revealed hemoglobin of 9.7 gm%, chest X ray suggested scoliotic deformity of dorsal spine with concavity to right side at T3 level, ECG showed right bundle branch block with left anterior hemiblock. ECHO revealed mild mitral regurgitation, tricuspid regurgitation, pulmonary artery hypertension with severe left ventricular dysfunction (ejection fraction of 30-35%). Pulmonary function test (PFT) results were ambiguous. She was on digoxin, ramipril, carvidilol, frusemide and spironolactone as advised by the cardiologist. The patient was tall and lean, had cachectic appearance. On examination she had a pulse rate of 100/min, blood pressure of 90/61 mm Hg, respiratory rate of 14/min and slight pallor. Evaluation of patient's airway revealed protruding teeth, normal mouth opening and adequate neck mobility. Her Mallampatti grade was II. There was no history of any previous hospitalizations except for undergoing 2 premature abortions 10 and 8 years back. Cardiologist was consulted about the patient's medical management preoperatively in the ward. Clearance was given for surgery under high risk consent and she was advised to continue her drugs as mentioned above. She was posted for exploratory laparotomy on semi emergency basis and was accepted as ASA IIIE. Patient and her relatives were explained about the anaesthetic and surgical risk. General anaesthesia (GA) with epidural anaesthesia was planned after a detailed explanation to the patient in the preoperative period. She was advised oral alprazolam 0.25 mg and ranitidine 150 mg at bed time night before surgery and on the morning of surgery. Inside the OT patient was connected to all routine monitors. Hemodynamic parameters were stable but oxygen saturation showed 91% on room air. Ventimask with FiO2 0.5 @ 5L/min was attached to the patient. An IV line and a 16 G peripheral CVP line (CVP=10 cm H2O) was inserted under LA (1% plain lidocaine). Difficult airway cart was prepared in view of suspected difficult intubation. Defibrillator with transcutaneous pacing facility was kept standby for any sudden arrhythmia. Patient was preloaded with 500 ml of lactated Ringer solution and the epidural catheter was inserted at L3-4 level in the left lateral decubitus position through an 18 gauge Tuohy needle and fixed at 9 cm at skin. Test dose of 3 ml of 2% lidocaine without adrenaline was administered. After preoxygenation, IV fentanyl 60 mcg and propofol 40 mg was given. Patient's lungs were ventilated with 50% O2 and 50% N2O and trachea was intubated with size 7.5 tube following IV atracurium 25 mg. Anaesthesia was maintained with 0.8% isoflurane in 50% O2, and 50% N2O and atracurium 10 mg repeated whenever required. Morphine 1.5 mg in 5ml was given through epidural catheter intraoperatively. There was no haemodynamic instability through out the surgical procedure with heart rate maintained between 74-78 beats per minute, and the blood pressure around 100/ 65 mm Hg. Surgery lasted 80 min with 250 ml blood loss. After surgery the patient was reversed with adequate doses of glycopyrrolate and neostigmine and shifted to post operative anaesthesia care suite. On the 2nd postoperative day patient developed respiratory distress and hypotension. Arterial blood gas analysis was done and suggested Type 1 respiratory failure. Ventimask with FiO2 0.5 was attached, followed by chest physiotherapy and nebulisation with salbutamol. The patient didn't improve with this treatment and had to be intubated and shifted to ICU. Chest X-ray was suggestive of pneumonia of left hemithorax so she was kept on SIMV mode of ventilation.

Despite not being on any muscle relaxant and sedatives the patient was tolerating the endotracheal tube and was sedated. Consultant anaesthesiologist then noticed fasiculations of facial muscles and head of the patient was turned continuously to one side suggesting sternocleidomastoid spasm. Myotonic dystrophy was suspected based on the history, findings of repeated muscle spasms, sternocleidomastoid spasm, cardiomyopathy and the abnormal facies i.e. temporal flattening and frontal balding. On enquiring from her husband it was revealed that she had a sister with a similar disorder. Opinion of a neurologist was sought and as advised by them muscle biopsy and muscle enzyme study were done. These revealed probable muscle disorder for which genetic analysis was done wherein CTG repeats were found in 3-prime-untranslated region of DMPK (160900) were found which maps to 19q13.3, suggestive of myotonic dystrophy. Since there is no definitive treatment of myotonic dystrophy the patient was given supportive therapy in ICU. Patient was tracheostomised on day 7 and due care was given to her nutrition and physiotherapy. She was intermittently given T piece trials but she could maintain on T piece for few hours only. Despite all possible efforts we were unable to wean her off the ventilator for 390 days. She had 3 episodes of cardiac arrest during her stay in ICU but was revived each time successfully. Ultimately she died of cardio-respiratory arrest on 391st day.

## Discussion

Myotonic dystrophy(MD), also referred to as Dystrophica myotonica or Steinert's disease, is an autosomal dominant degenerative condition. It is the most common and most severe of the myotonic syndromes with a prevalence of 3-5 in 100,000 worldwide. It results from a defective gene located on chromosome 19 wherein there is expansion of CTG trinucleotide repeat in the gene DM protein kinase. The disease is diagnosed by molecular genetic testing. It has three phenotypes: 1. Mild MD: Patients present with mild myotonia and cataract. 2. Classic MD. Patients present with severe muscle weakness, myotonia and cardiac abnormalities, all resulting in increased morbidity. 3. Congenital MD: Neonates present with hypotonia at birth, mental retardation and respiratory insufficiency.^1^,^2^ Morphological features include frontal baldness, hatched face, ptosis, wasted sternocleidomastoid (SCM) and masseter muscles and distal muscle atrophy of the upper and lower limbs. On analysis we can say that our patient was probably a case of Classic MD and had cardiomyopathy, wasting of limb muscles; facial muscle fasiculations, SCM spasm, infertility, typical hatched faced appearance. Anaesthetic management of these patients is challenging and may pose a serious problem to the anaesthesiologist. Hypothermia, shivering, and mechanical or electrical stimulation may precipitate myotonia. Moreover, they are sensitive to sedative, anaesthetic and neuromuscular blocking agents, which may result in intraoperative and early postoperative cardiovascular and respiratory complications, as well as prolonged recovery from anaesthesia.^2^,^3^ Our management plan was dedicated primarily towards the cardiac condition. GA carries a high risk as these patients may develop CHF or arrhythmias during intraoperative period. Regional anaesthesia may be an alternative to general anaesthesia in selected patients with DCM. Epidural anaesthesia produces changes in the preload and afterload that mimic pharmacological goals in the treatment of this disease Among inhalational and IV anaesthetic agents, low dose ketamine has least direct negative inotropic action on myocardium in patient with ischemic DCM with low EF. IV midazolam and fentanyl can cause hypotension but titrated low dose has minimal effect. Propofol and thiopentone have unacceptably high risk of causing hypotension in patient with DCM. For that reason we used drugs in titrated manner for appropriate level of conscious sedation and were ready for any accidental event.

In our case we did not think of myotonic dystrophy as a cause of the patient's general physical and cardiac conditions. Since incidence of MD is low and this was first case we had ever seen, we missed the diagnosis in our patient. Respiratory failure is a frequent cause of postoperative morbidity because weakness of pharyngeal muscles may lead to aspiration pneumonia which was probably what happened in our patient. Weakness of diaphragm and accessory muscles of respiration and low central ventilatory drive often predisposes them to alveolar hypoventilation leading to atelectasis.^1^,^2^ Moreover these patients are very sensitive to both IV and epidurally administered opiates 4.Epidural morphine must have precipitated hypoventilation and subsequent pneumonia. Cardiac involvement is common with progressive deterioration in the conducting system resulting in heart block and sudden death. In anaesthesia for patients with MD one should avoid using both depolarizing and nondepolarising muscle relaxants 5,6 and prefer regional anaesthesia alone or general in combination with regional. If use of muscle relaxants is necessary then one should use short acting nondepolarising muscle relaxants with NM monitor. Anticholinesterase agents may precipitate myotonic crisis and severe bradyarrythmias in MD patients.^6^ Residual curarisation should be treated with mechanical ventilation. Sedatives should be used cautiously. Though complications are common in MD but there are a lot of reports 1,2,5 of patient being alive after successful administration of anaesthesia. One should suspect MD on basis of morphological and previous features. Risk of perioperative complications is increased in MD. Meticulous care of MD patients before, during and after surgical intervention is strongly recommended to avoid complications.
